# Foolish Use of Carbolic Acid

**Published:** 1890-03

**Authors:** 


### FOOLISH USE OF CARBOLIC ACID.

Prof. Billroth, of Vienna, warns once more against the imprudent use of carbolic acid as follows:

In the last month four cases have come to my notice where fingers with insignificant wounds have become gangrenous by foolish application of carbolic acid. All that four cases were children whose parents had prescribed a carbolic acid bandage by their own authority, because carbolic acid was said to be good for healing wounds. The use of carbolic acid is much more limited in surgery than before; it is only by degrees we have known the dangers it may present. This remedy may not only cause inflammations and gangrena, it may kill by blood-poisoning. Its good qualities are developed only in the hand of a competent ghysician. I dissuade most emphatically the application of carbolic acid without the prescription of a physician. I recommend as the best bandage for fresh wounds acetate of lead (lead-water), which is for sale in all drug stores. —*Pharmaceutische Post.*

